# A Comprehensive Survey of miRNA Repertoire and 3′ Addition Events in the Placentas of Patients with Pre-Eclampsia from High-Throughput Sequencing

**DOI:** 10.1371/journal.pone.0021072

**Published:** 2011-06-22

**Authors:** Li Guo, Qi Yang, Jiafeng Lu, Hailing Li, Qinyu Ge, Wanjun Gu, Yunfei Bai, Zuhong Lu

**Affiliations:** 1 State Key Laboratory of Bioelectronics, School of Biological Science and Medical Engineering, Southeast University, Nanjing, China; 2 Key Laboratory of Child Development and Learning Science of Ministry of Education, Southeast University, Nanjing, China; Istituto Dermopatico dell'Immacolata-IRCCS, Italy

## Abstract

**Background:**

To gain insight into potential roles of isomiR spectrum and isomiRs with 3′ additions in pre-eclampsia, we performed a comprehensive survey of miRNA repertoire and 3′ addition events from placental samples with different degrees of pre-eclampsia by applying SOLiD sequencing platform.

**Principal Findings:**

Over 30% isomiRs were detected with 3′ non-template additional nucleotides, especially for additional nucleotide of adenosine. However, these modified isomiRs showed a lower percentage of total miRNA expression (<15%). Generally, 1-3 abundant isomiRs from a given miRNA locus were identified, but none of them was detected with 3′ additions. Different miRNAs indicated various isomiR spectrums and expression patterns. The most abundant isomiR spectrum, isomiR profile and expression pattern always were stability, but herein we found several exceptions across samples, especially between normal and diseased samples. At isomiR level, we detected a distinct subset of differentially expressed modified isomiRs between normal and diseased samples or between mild and severe samples. Gene Ontology analysis of their experimentally validated target genes revealed enrichment for specific biological process categories.

**Conclusions:**

The phenomenon of multiple isomiRs, especially for isomiRs with 3′ additions, is not a random event during pre-miRNA processing. Varieties of isomiRs and expression patterns reveal potential functional implication and should be taken into account. The study enriches association of miRNAs and human disease, including potential roles of various miRNA variants and 3′ addition events.

## Introduction

MicroRNAs (miRNAs), a class of endogenous small non-coding RNAs (∼22 nt), play pivotal post-transcriptional regulatory roles in normal physiological functions by targeting messenger RNAs for cleavage or translational repression [1], [2], [3]. It is believed that miRNA contributes to regulatory network by complementary binding of its “seed sequence” (nucleotides 2–8) and targets in the 3′ untranslated region of mRNAs [4]. Altered expression of specific miRNAs has been reported to associate with a number of diseases, including various human cancers. Entire repertoires of miRNAs in normal and cancer tissues are performed to assess a subset of differentially expressed miRNA species and discover potential diagnostic miRNA biomarker. The small non-coding RNA is generated from a ∼70 nt miRNA precursor hairpin (pre-miRNA) that is resulted from a ∼1–3 kb primary miRNA transcript [5], [6]. Typical miRNA biogenesis shows that miRNA precursor produces active miRNA and inactive miRNA* sequences. miRNA, known as “mature miRNA”, is loaded into AGO to contribute regulatory network, whereas miRNA*, also termed as a passenger strand, is long thought to be degraded and discarded [7]. But recent evidence indicates that miRNA* can also be loaded into AGO2 and bind target mRNAs to play a role in regulation network as a potential regulatory molecule [8], [9], [10], [11], [12], [13].

High-throughput sequencing technologies are offering a broad and deep survey of the interesting and pivotal small non-coding regulatory molecules. Discovering and profiling of miRNAs have been widely studied in animals and plants, especially for various human diseases. Based on high-sensitivity and high-throughput sequencing datasets, more small RNAs are detected, including multiple miRNA variants (isomiRs) [14], [15], [16], [17], [18] and microRNA-offset RNAs (moRNAs) [19], [20], [21]. Multiple isomiRs with various 5′ and/or 3′ ends are thought to be the result of inexact Drosha and Dicer processing. These small RNAs have greatly enriched study of miRNAs and broadened complex regulation network. Moreover, the phenomenon of 3′ addition events, especially for post-transcriptional non-template 3′ end additions of adenosines or uridines, is widely detected in animals and plants [4], [14], [17], [22], [23], [24], [25], [26], [27], [28], [29], [30]. In animals, these terminal nucleotides are added after Dicer processing [31], and most 3′ additions are added to canonical miRNA sequences (reference miRNA sequences in the miRBase database) [24]. The phenomenon is not a random event, but is widespread and conserved across animal species [23]. Further, 3′ additions may contribute to miRNA stability and play a role in interactions of miRNA:target [23], [24]. Multiple isomiRs with heterogeneous ends and non-template nucleotide additions are differentially expressed across different development and tissues in *Drosophila melanogaster* [24]. 3′ addition events show potential biological function in complex regulation network. For example, isomiRs with 3′ additions may increase miRNA stability in *Drosophila* [24], may be differentially loaded into Argonautes [4], [14], may attenuate the effectiveness of specific miRNAs [23], and isomiRs with adenosines are less prone to degradation in *P. trichocarpa* [28]. However, little is known about whether there is a potential relationship between multiple isomiRs and human disease.

Pre-eclampsia is a relatively common human disease of pregnancy characterized by hypertension and proteinuria. It originates in the placenta and causes some maternal and fetal problems, and even might threaten maternal and perinatal survival. Abundantly and differentially expressed miRNA species in placental samples were reported by applying microarray analysis and real-time quantitative reverse transcription-ploymerase chain reaction [32], [33], [34], [35], [36], [37], [38], [39], but little is known about isomiR profile and potential association with different degrees of human pre-eclampsia. In the study, to test whether multiple isomiRs and 3′ addition events are biologically regulated and show close relationship with different degrees of pre-eclampsia, we deep-sequenced small RNA libraries by applying the next generation high-throughput sequencing platform, SOLiD System (ABI, Life Technologies). Based on a comprehensive analysis, multiple miRNA variants and miRNAs with 3′ modifications were identified and profiled across placental samples. Simultaneously, due to multiple isomiRs from a given locus with various expression levels, we therefore assessed miRNA profile by employing the most abundant isomiR and sum of all isomiRs, respectively. Differentially expressed miRNAs and isomiRs with 3′ additions were surveyed and further studied across different samples.

## Results

### A widespread phenomenon of 3′ addition events

**Figure 1:**
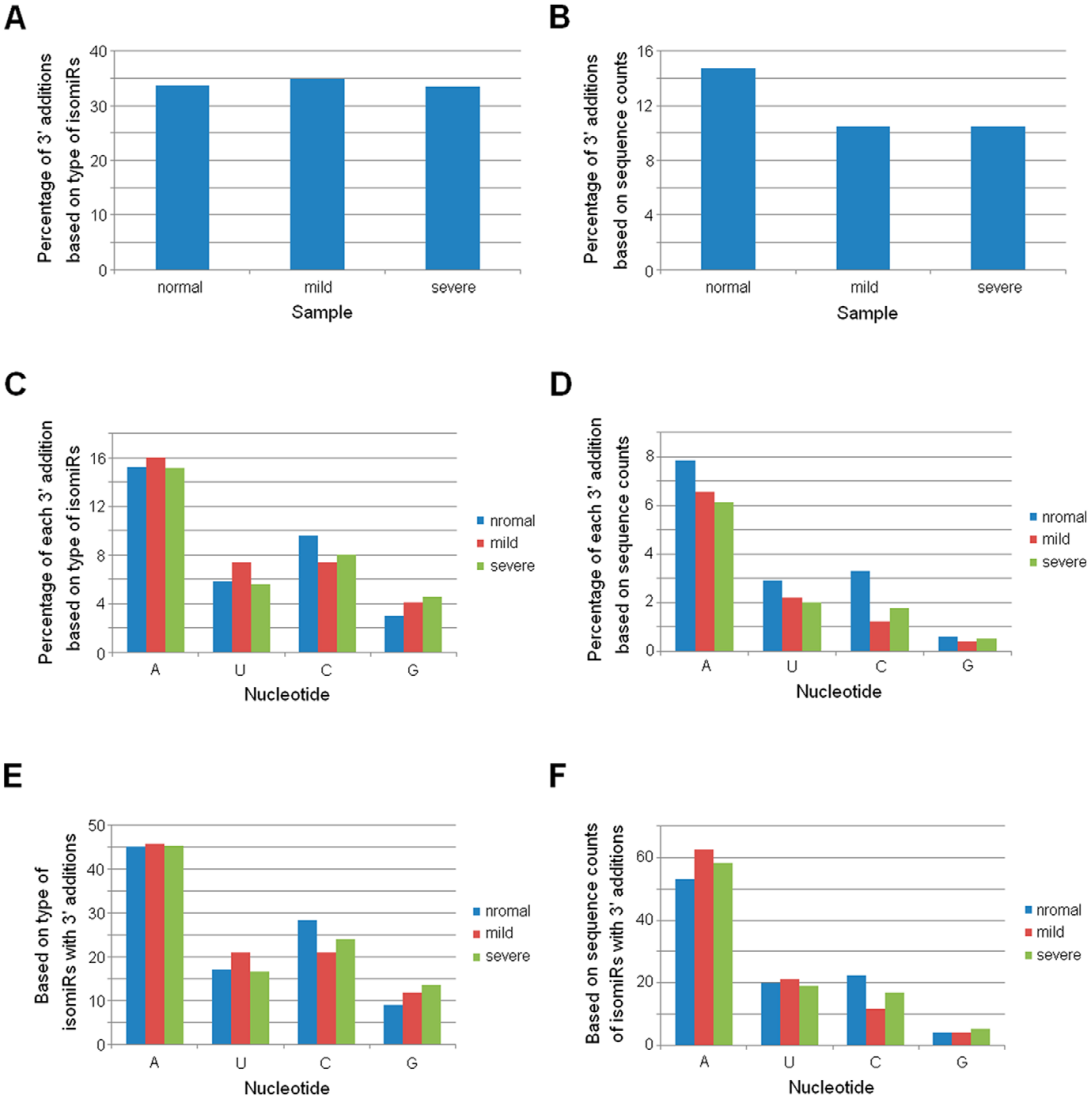
Percentage distributions of isomiRs with 3′ additions. IsomiRs are estimated if their sequence counts are not less than 10. Percentage of 3′ additions based on (A) type of isomiRs; (B) sequence counts of all isomiRs; (C) type of all isomiRs; (D) sequence counts of all isomiRs; (E) type of isomiRs with 3′ additions; (F) sequence counts of isomiRs with 3′ additions.

Using the next generation sequencing technology, SOLiD System, we sequenced the small RNAs of placental samples from pregnant women with mild and severe pre-eclampsia and normal pregnant woman (Table S1). According to short RNAs that could be mapped to human miRNA precursors, the most abundant length was 22 nt, as expected (Figure S1). Although similar length distribution patterns were detected across the three samples, different percentages could be found, especially between normal and diseased samples (Figure S1). miRNAs with 3′ non-template additional nucleotides were widely detected in the three samples, and adenosine was the most abundant and prevalent additional nucleotide (Figure 1). Over 30% isomiRs were found to have 3′ additions (Figure 1A). Although there was much larger percentage based on type of isomiRs, these modified isomiRs showed lower percentage of total expression level (lower than 15%, Figure 1B). Compared with diseased samples, isomiRs with 3′ additions in normal sample showed higher percentage of total miRNA expression (about 14.74%). Over 15% total isomiR types were characterized by 3′ additional of adenosine (Figure 1C and Figure 1D). Additional cytosine was more prevalent in normal control sample than diseased samples (Figure 1C–F). Rare 3′ additional guanine was detected in the three samples.

**Figure 2:**
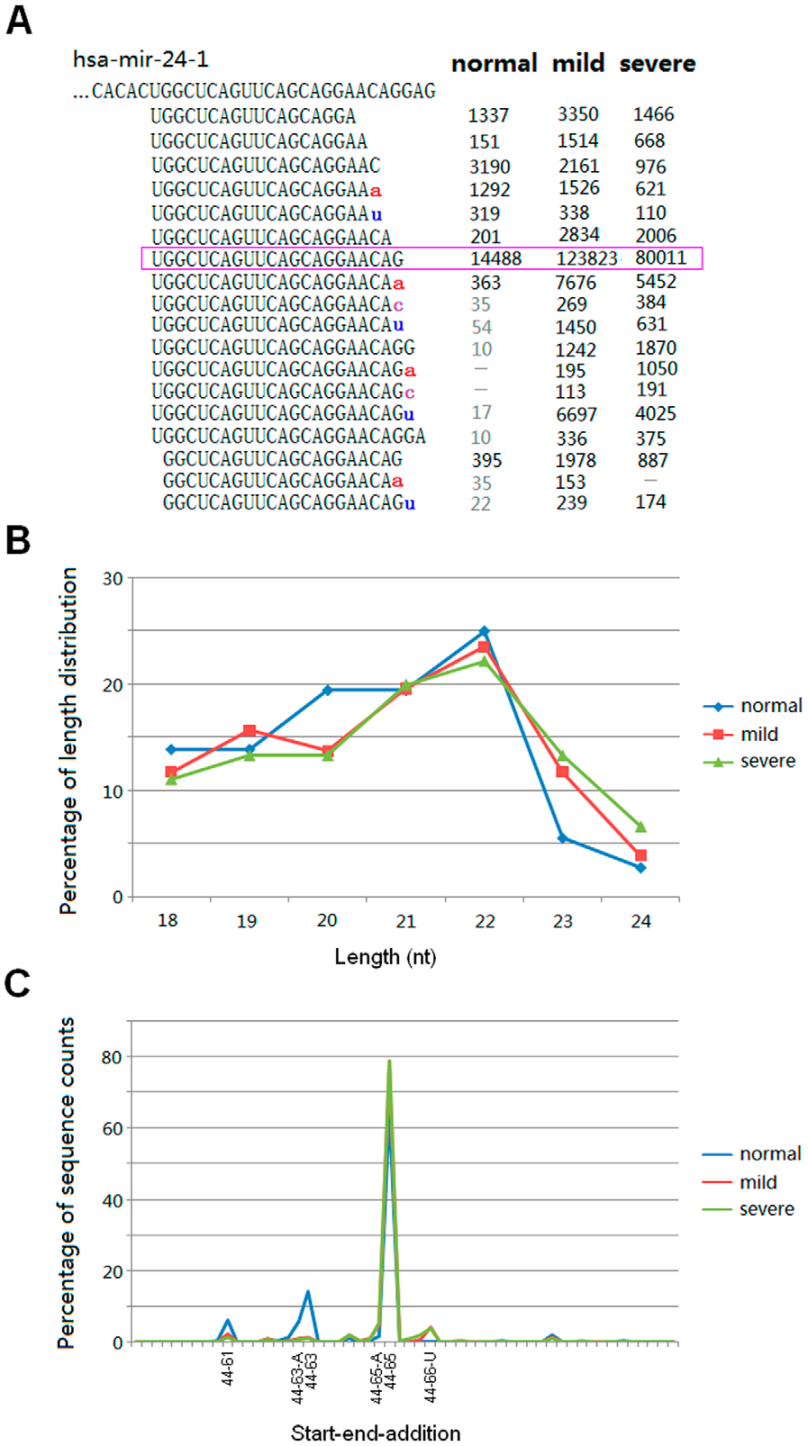
Detailed isomiRs, their length distributions and relative expression levels. Hsa-miR-24 is the most abundant miRNA in the three samples. (A) Detailed isomiRs and their sequence counts across samples. Sequence in pink box is canonical hsa-miR-24 sequence in the miRBase database. 3′ additions are highlighted by using lowercase letters in different colors. IsomiRs are not presented here if they have lower sequence counts than 100 in mild and sever samples. The signal of “—” shows sequence count of the isomiR is lower than 5 or not detected in normal sample. (B) Length distribution of isomiRs based on their sequence counts. As expected, the most dominant length is 22 nt. (C) Relative expression levels of isomiRs are estimated based on percentage of all isomiRs of hsa-miR-24. These isomiRs are showed according to start and end sites on hsa-mir-24-1 sequence. Evident differences can be detected of isomiRs with locations of 44–61, 44–63-A, 44–63 and 44–65-A.

IsomiRs with 3′ non-template additional nucleotides may have shorter, longer or consensus lengths with their canonical miRNA sequences. For example, modified isomiRs of hsa-miR-24 showed various length distributions, and additional nucleotide could be added 3′ end of canonical miRNA sequence, shorter or longer isomiRs (Figure 2). Although some isomiRs with 3′ additions also had higher sequence counts, they were not abundant isomiR species in the miRNA locus (Figure 2).

### The top 10 abundant miRNA species and modified isomiR species

**Figure 3:**
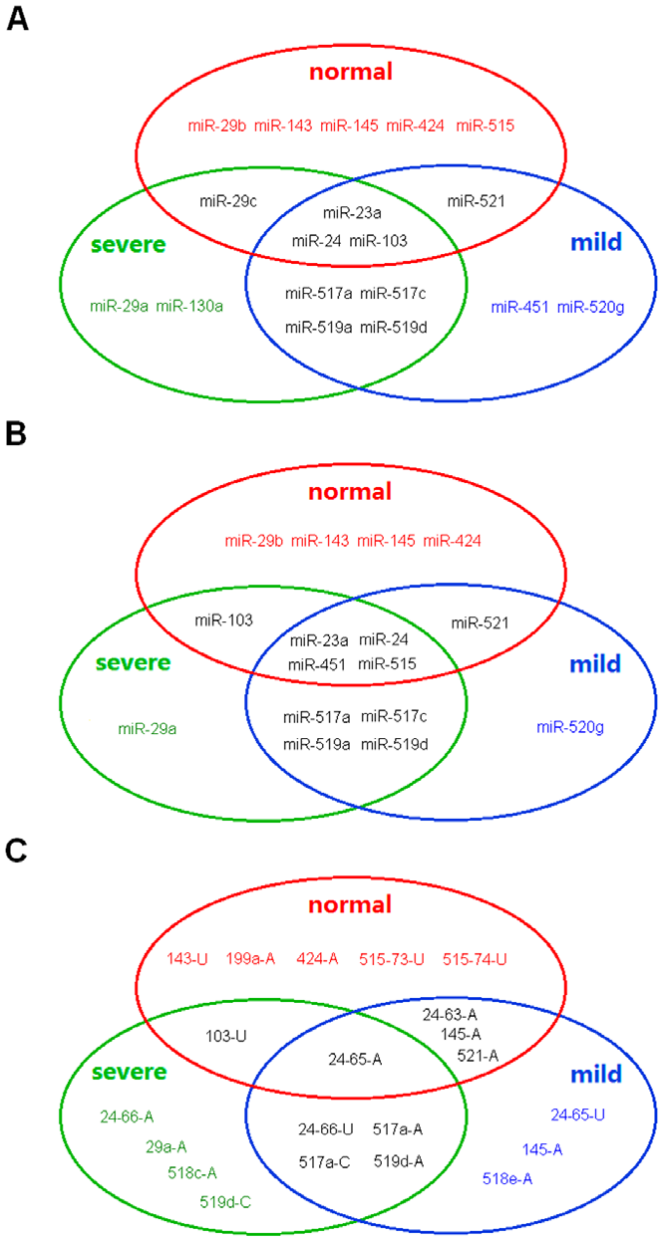
Distribution of abundantly expressed miRNAs across samples. The top 10 abundant miRNA species are estimated based on (A) the most abundant isomiR and (B) sum of all isomiRs. The top 10 abundant isomiR species with 3′ additions are also estimated (C).

According to the sequence count of the most abundant isomiR from a single miRNA locus, we assessed the top 10 abundant miRNA species (Table 1). Hsa-miR-24 was the most abundant miRNA, while only 3 miRNAs were shared by the three samples (Table 1 and Figure 3A). There were 7 common miRNAs between mild and severe samples, but they showed different order distributions (Table 1). Simultaneously, we also reassessed the top 10 abundant miRNA species according to sum of all isomiRs. Similar to Guo & Lu (2010) [40], the inconsistent order distribution was shown based on different estimation methods (Table 1). We therefore obtained another subset of the top 10 abundant miRNA species based on sum of all isomiRs, as expected (Figure 3B). Similar distribution patterns were detected based on different estimation methods despite involved different miRNA species (Figure 3A and Figure 3B). Mild and severe samples shared more common miRNAs than between normal control and diseased samples (Figure 3A and Figure 3B).

**Table 1:** The top 10 most abundant miRNA species in the three samples.

No.	Normal		Mild		Severe	
	The most	All isomiRs	The most	All isomiRs	The most	All isomiRs
1	miR-24	miR-24	miR-24	miR-24	miR-24	miR-24
2	miR-521	miR-515	miR-517a	miR-517a	miR-517a	miR-517a
3	miR-29b	miR-145	miR-519a	miR-519d	miR-519d	miR-519d
4	miR-103	miR-143	miR-519d	miR-519a	miR-519a	miR-519a
5	miR-515	miR-424	miR-517c	miR-23a	miR-103	miR-23a
6	miR-424	miR-521	miR-521	miR-451	miR-29a	miR-103
7	miR-143	miR-29b	miR-23a	miR-520g	miR-130a	miR-29a
8	miR-145	miR-103	miR-103	miR-517c	miR-517c	miR-517c
9	miR-23a	miR-23a	miR-520g	miR-521	miR-23a	miR-130a
10	miR-29c	miR-29c	miR-451	miR-103	miR-29c	miR-29c

The top 10 most abundant miRNA species are estimated based on the most abundant isomiR (The most) and sum of all isomiRs (All isomiRs), respectively.

The top 10 most abundant isomiR species with 3′ additions showed that some of them were yielded from the same miRNA locus (Table 2). For example, several of them were modified isomiRs of hsa-miR-24 (Table 2 and Figure 2A). We found many abundant modified isomiRs had the same lengths with their canonical miRNA sequences, which indicated 3′ non-template nucleotides were added to the 3′ ends of shorter isomiR sequences than canonical sequences (Table 2 and Figure 2). It is inconsistent with a previous report that isomiRs with 3′ additions always were longer than canonical miRNA sequences [24]. Indeed, the shorter isomiR always also showed higher expression level, and even was the most abundant isomiR (for example, the length of the most abundant hsa-miR-145 in normal sample was shorter than its canonical length). The particular phenomenon was more prevalent in diseased samples than normal sample (Table 2). Compared with the top 10 abundant miRNA species, perhaps due to diversity of isomiR species with 3′ additions, less common isomiRs were detected across the three samples (Figure 3). We only found one common isomiR among normal and diseased samples (hsa-miR-24–65-A, “65” indicated the end site on hsa-mir-24-1). There were 3 common isomiRs between normal and mild samples, while only hsa-miR-103-U was shared by normal and severe samples (Figure 3C).

**Table 2:** The top 10 most abundant isomiR species with 3′ additions in the three samples.

No.	Normal		Mild		Severe	
	miR-addition	Start-end (ref)	miR-addition	Start-end (ref)	miR-addition	Start-end (ref)
1	miR-24-A	44–63 (44–65)	miR-24-A	44-65	miR-24-A	44–65
2	miR-143-U	61–82 (61–81)	miR-24-U	44–66 (44–65)	miR-24-U	44–66 (44–65)
3	miR-424-A	11–33 (11–32)	miR-519d-A	54–76 (54–75)	miR-519d-A	54–76 (54–75)
4	miR-145-A	16–36 (16–38)	miR-517a-A	54–75	miR-24-A	44–66 (44–65)
5	miR-515-U	51–74 (51–72)	miR-518e-A	54–75 (54–74)	miR-517a-A	54–75
6	miR-103-U	48–70	miR-24-A	44–63 (44–65)	miR-517a-C	54–75
7	miR-515-U	51–73 (51–72)	miR-24-U	44–65	miR-518c-A	62–84
8	miR-24-A	44–65	miR-517a-C	54–75	miR-103-U	48–70
9	miR-199a-A	47–69 (47–68)	miR-145-A	16–36 (16–38)	miR-29a-A	42–64 (42–63)
10	miR-521-A	54–75	miR-521-A	54–75	miR-519d-C	54–76 (54–75)

“Start-end (ref)” shows start-end sites on corresponding precursor including 3′ non-template nucleotide, and “ref” shows reference start-end sites on the precursor in the miRBase database. Empty “ref” shows consistent location. If specific miRNA has multicopy precursors, we here default the first miRNA precursor.

### Differentially expressed miRNAs and modified isomiRs

**Figure 4:**
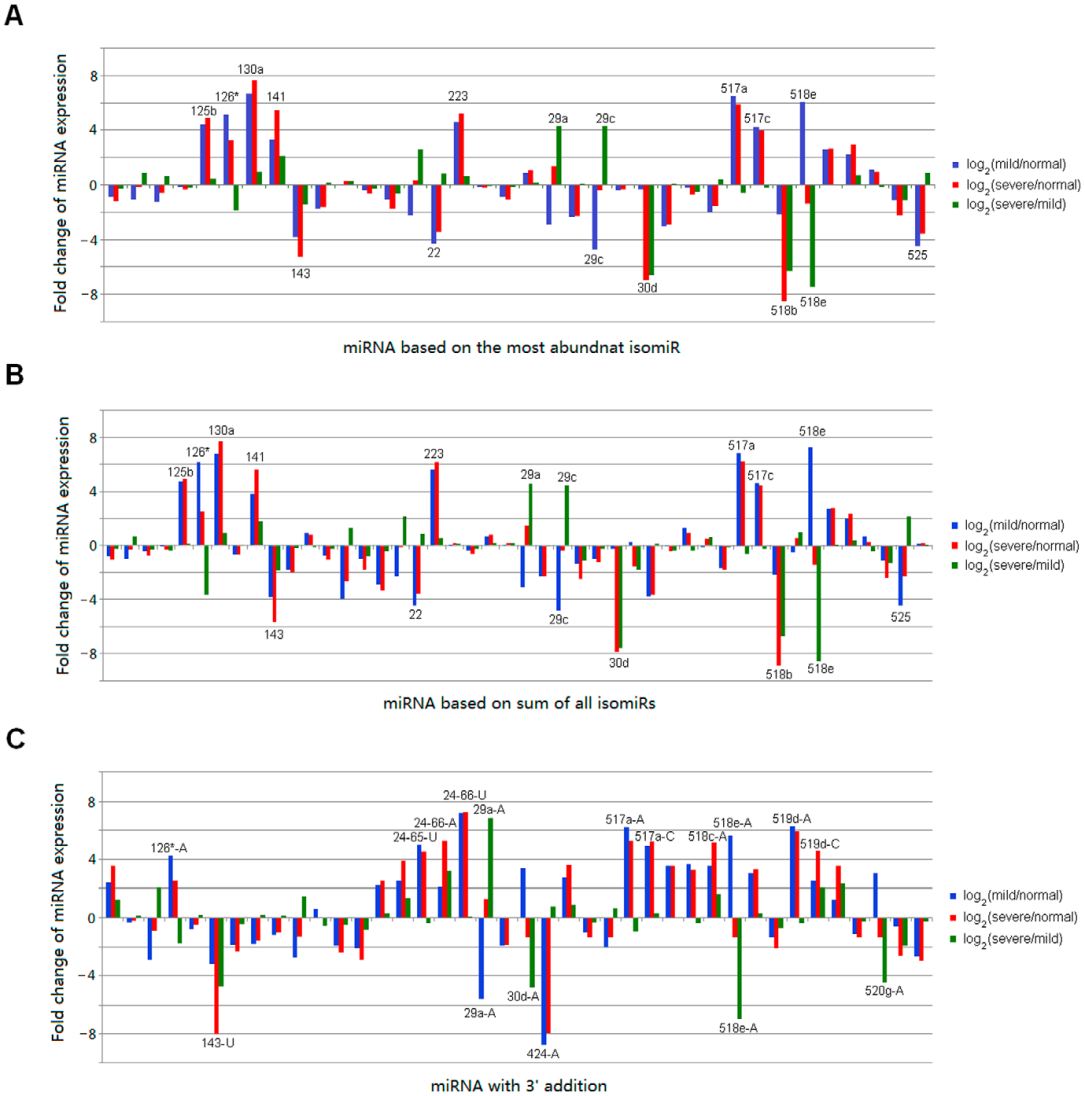
Differentially expressed miRNAs and modified isomiRs across samples. Fold change of miRNA expression of pairwise samples is estimated (A) sequence count of the most abundant isomiR (>999 at least in one sample), (B) based on sum of all isomiR sequence counts (>999 at least in one sample) and (C) isomiR with 3′ addition (>300 at least in one sample), respectively. If fold change value is more than 4 or lower than -4, the differentially expressed miRNA will be marked. If some miRNAs are not detected or their sequence counts are less than 100, 10 will be their default sequence counts.

Different estimation schemes were used to assess differentially expressed miRNAs across different samples. According to sequence count of the most abundant isomiR, fold change values of 15 miRNAs were over 4 or less than -4 between at least one pair of samples (Figure 4A). Almost all of them were differentially expressed between normal and diseased samples, and 5 of them were detected between mild and severe pre-eclampsia samples (Figure 4A). The same miRNA species could be detected if sum of all isomiR sequence counts were used to assess expression profiles (Figure 4A and Figure 4B). However, fold change values might be slightly changed based on different estimation methods. For example, log_2_(severe/normal) of hsa-miR-143 was −5.29 (based on the most abundant isomiR) and −5.69 (based on sum of all isomiRs), respectively.

Meanwhile, at isomiR level, we also estimated differentially expressed isomiRs with 3′ additions. A total of 40 abundant modified isomiRs were analyzed, and 15 of them were differentially expressed at least between one pair of samples (Figure 4C). Similarly, many of these isomiRs were detected between normal and diseased samples, and only 5 of them were detected between mild and severe samples (hsa-miR-143-U, 29a-A, 30d-A, 518e-A and 520g-A). Among these differentially expressed modified isomiRs, seven of them were consistent with differentially expressed miRNA species (Figure 4). Overall, most of these abundant 3′ additional nucleotides were adenines, and no guanines were detected (Figure 4C). Interestingly, except for isomiR of hsa-miR-520 g, other isomiRs with 3′ additions had the same 5′ ends and “seed sequences” with their canonical miRNA sequences in the miRBase database. Thus, we collected their experimentally validated targets sites from the miRTarBase database (Table S2). Gene Ontology analysis of these experimentally validated target genes revealed enrichment for specific biological process categories, for example, regulation of transcription, apoptosis, cell cycle, immune response, response to stimulus, etc.

### Varieties of isomiR spectrums and expression patterns

**Figure 5:**
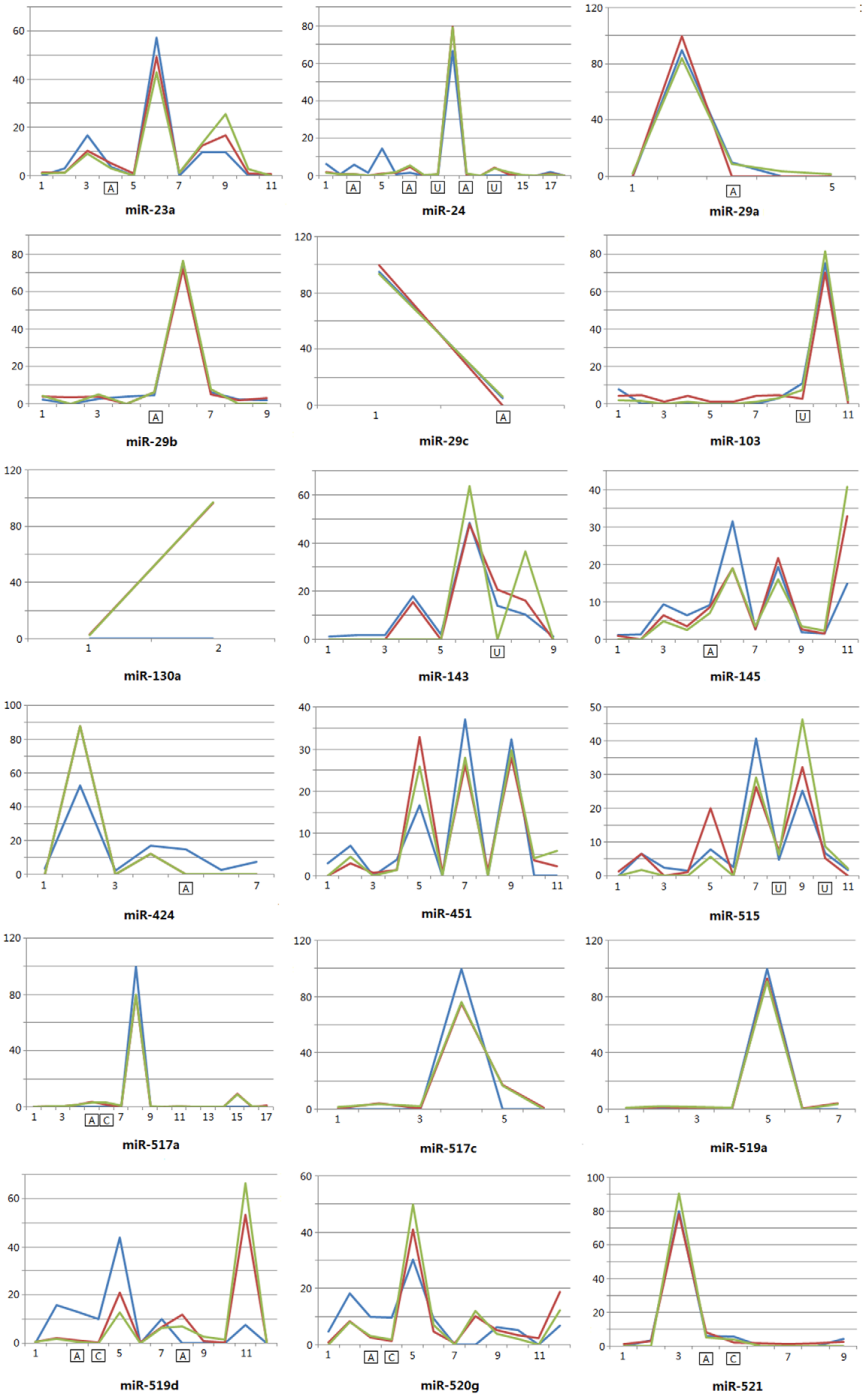
Various of type of isomiRs and their expression patterns across samples. The ordinate axis indicates percentage expression, and the horizontal axis indicates type of isomiRs from a given miRNA locus. If modified isomiR shows over 5% expression percentage or had higher sequence count (>999 in at least one sample), it is highlighted in the horizontal axis using the additional nucleotide. Blue line indicates isomiRs in normal sample; red line indicates isomiRs in mild sample; green line indicates isomiRs in severe sample. Relative expression levels are estimated based on percentage of sequence counts. All of these miRNAs are identified as the top 10 abundant miRNAs at least in one sample.

**Figure 6:**
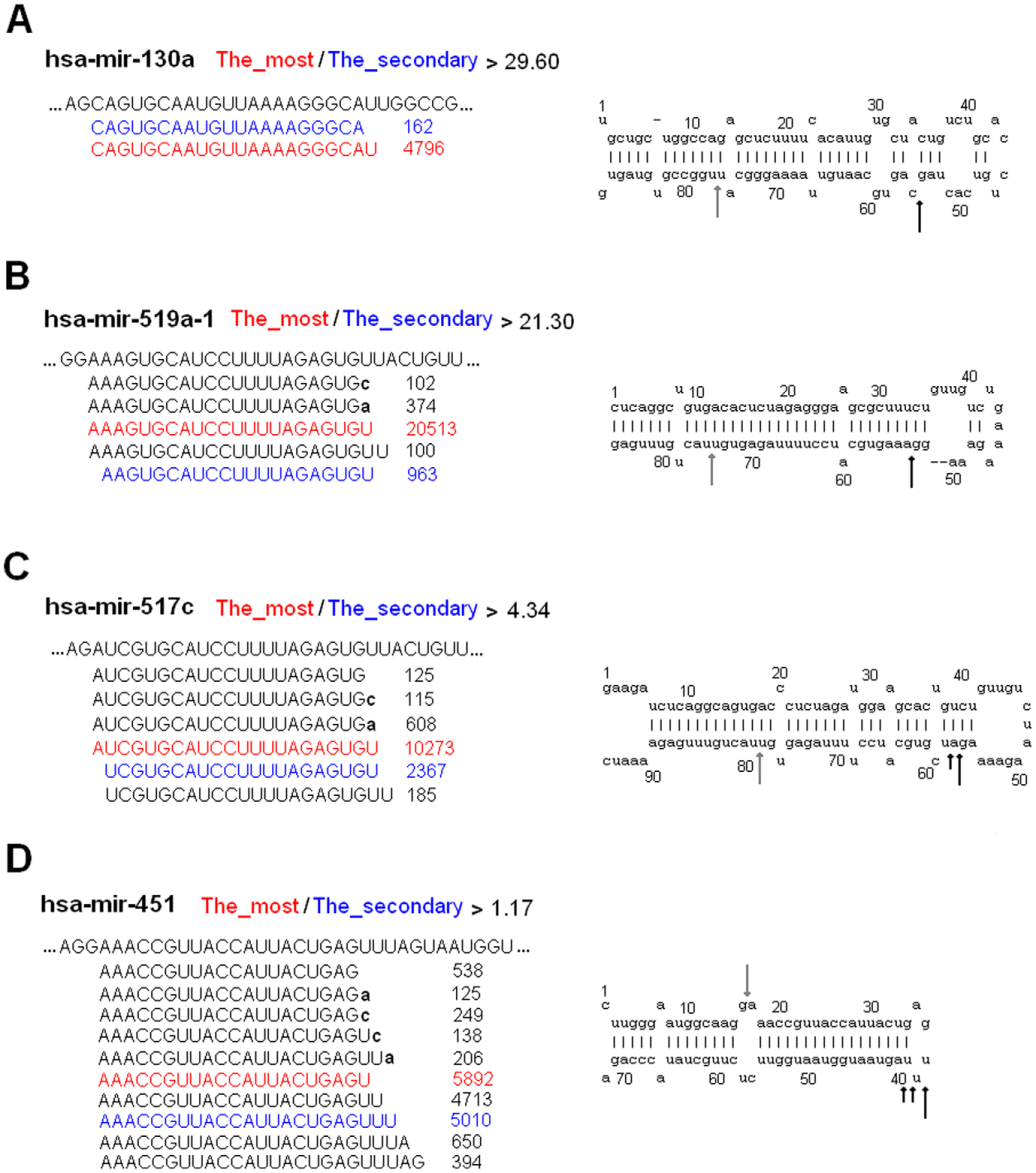
Multiple isomiRs through alternative and imprecise cleavage. Part of miRNA precursor, multiple isomiRs (sequence counts >99) with their sequence counts and second structure of pre-miRNA with dominant cleavage sites are presented here. Sequence in red background is the most abundant isomiR, while sequence in blue is the secondary abundant isomiR. 3′ non-template nucleotides are highlighted by using lowercase in black body. Inferred dominant cleavage sites of Drosha and Dicer are indicated in grey and dark arrows, respectively. If the fold of the most and the secondary abundant is less than 5, non-dominant cleavage sites are indicated using small arrows.

**Figure 7:**
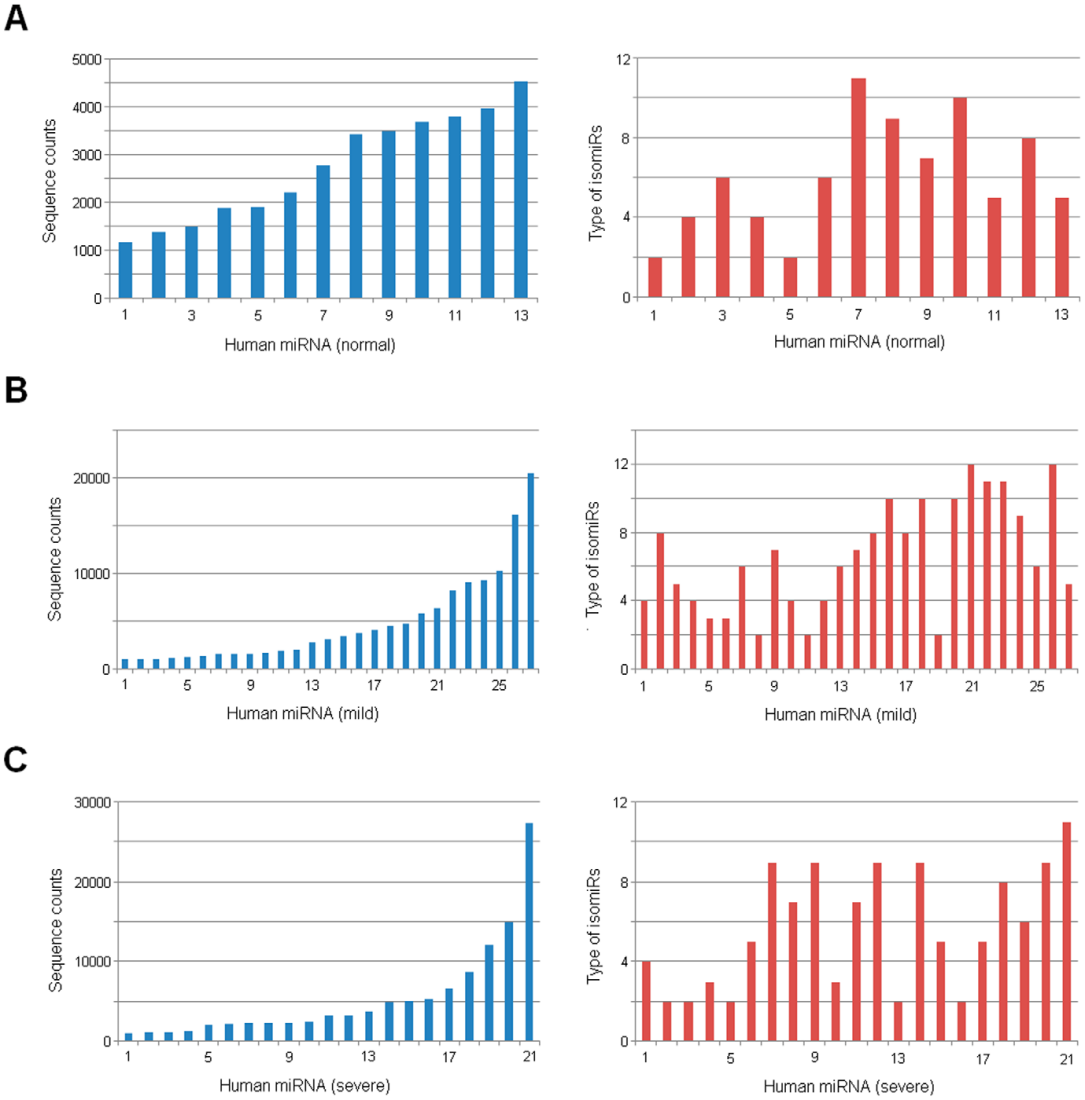
Distribution patterns of miRNAs and type of isomiRs. Expression distribution of miRNAs is assessed from lower to higher expression levels based on the most abundant isomiR, while their corresponding types of isomiRs show chaos distributions. All of these miRNAs are abundantly expressed in corresponding sample (sequence count of the most abundant isomiR is over 999). Type of isomiRs is assessed based on their sequence counts (>99).

Multiple isomiRs with various 5′ and/or 3′ ends and expression levels, including isomiRs with 3′ additions, were detected from a given miRNA locus (Figure 2 and Figure 5). Despite involved various 5′ and/or 3′ ends due to imprecise cleavage of Drosha and Dicer, variation at 3′ ends was more prevalent than 5′ ends (Figure S2). Generally, 5′ ends were more conserved than 3′ ends, which ensured stability of their “seed sequences” (nucleotides 2-8). Different miRNAs showed different numbers of miRNA variants with various expression levels. For example, in mild sample, miR-451 was found 10 variants (sequence counts of them were over 99), while miR-130a was only found 2 variants (Figure 5 and Figure 6). Although expression levels of the two miRNAs were similar, their isomiR types and expression patterns showed a distinct difference. Moderate correlation was reported between expression level of miRNA and type of isomiRs [15], but unexpectedly, we herein found some exceptions: miR-519a was found fewer type of isomiRs although it had higher expression levels than miR-451 (Figure 6). To obtain more detailed correlation between expression level of miRNA and type of isomiRs, we performed a comprehensive analysis according to abundant miRNA species by employing the most abundant isomiR and sum of all isomiRs, respectively. No strict correlation was detected, especially based on the most abundant isomiR (Figure 7, Figure S3 and Table S3).

Interestingly, some miRNAs showed inconsistent the most abundant isomiR spectrums (for example, hsa-miR-145, 451, 515 and 519d) across the different samples, especially between normal and diseased samples (Table S4). Despite of the difference in sequences, these isomiRs were 3′ isomiRs with the same 5′ ends. They may be canonical miRNA sequences or inconsistent sequences. In order to further understand isomiR spectrum, we also estimated fold change of the most and secondary abundant isomiRs. Except for hsa-miR-519d, fold changes always were less than 2, which suggested several abundant isomiRs with similar expression levels from a given miRNA locus (Table S4). Indeed, the phenomenon of various expression differences could be detected according to more miRNAs: the most abundant isomiR of miR-130a was over 29.60-fold than the secondary abundant isomiR (Figure 6A), while similar expression levels (over 1.17-fold) could be found between the most and secondary abundant isomiRs of miR-451 (Figure 6D). According to expression distributions of multiple isomiRs and fold change values of the most and secondary abundant isomiRs, we also estimated dominant cleavage sites of Drosha and Dicer during pre-miRNA processing (Figure 6). Dominant cleavages sites always were concentrated on some specific sites (1–3 continuous sites) (Figure 6).

Although multiple isomiRs with various 5′ and/or 3′ ends from a given locus were identified, they showed various expression levels. Generally, 1–3 isomiRs were abundantly expressed, whereas others always had fewer sequence counts (Figure 5). Strikingly, although some isomiRs with 3′ non-template nucleotides had higher sequence counts, they only showed a very low percentage of total expression from the given locus (Figure 2 and Figure 5). The abundant isomiRs were canonical miRNA sequence or inconsistent sequences without 3′ additions. Similar expression distributions could be detected across the three samples, especially some of them indicated consensus distributions (for example, hsa-miR-519a and hsa-miR-521). Based on a single miRNA, relative expression level of specific isomiR might be different, especially between normal and diseased samples. For example, although similar expression distributions of isomiRs of hsa-miR-517c were detected, specific isomiR showed different percentage of total expression (Figure 5).

## Discussion

### Non-random isomiR spectrum reveals biological implication

Due to high-sensitivity of high-throughput sequencing technologies, multiple miRNA variants with heterogeneous ends, lengths and expression levels, termed as isomiRs, were widely detected in animals and plants [14], [15], [16], [17], [18]. Although abundantly expressed miRNA has the potential to generate more type of isomiRs, and type of isomiRs shows a moderate correlation with expression level of corresponding miRNA [15], we herein found that there was no strict connection between them (Figure 6, Figure 7, Figure S3 and Table S3). Expression level of miRNA sometimes was not a critical factor in generating type of isomiRs (Figure 6). This phenomenon indicated complexity during processing of pre-miRNAs (Figure 7, Figure S3 and Table S3). Type of isomiRs from a given locus, particularly for those abundant isomiRs, always was conserved across different samples, but they also showed flexibility due to involved differences of total sequencing reads (Figure 5, Table S1 and Table S3). Despite more or less type of isomiRs was detected, significant expression differences were easily detected. Generally, every miRNA locus yielded 1–3 abundant isomiRs and other rare isomiRs (Figure 2, Figure 5 and Figure 6). Therefore, expression differences of isomiRs led to various fold changes of the most and secondary abundant isomiRs. Dominant cleavage sites of Drosha and Dicer were estimated based on the fold changes, and distinct differences among different miRNAs were found (Figure 6). The dominant cleavage sites always were concentrated on some specific regions (1–3 continuous sites), and therefore generated several abundant isomiRs (Figure 6). These abundant isomiRs always were 3′ isomiRs with the same 5′ ends and “seed sequences”, but none of them was detected with 3′ non-template additional nucleotides. The interesting dominant cleavage ensured consistent target sites.

More importantly, we found bias of the degree of heterogeneity between 5′ and 3′ ends based on a comprehensive analysis without involved isomiRs with 3′ additions (Figure S2). 3′ isomiRs with various 3′ ends were more prevalent than 5′ isomiRs, and abundantly expressed isomiRs always were 3′ isomiRs. It is well known that miRNAs will involve new identities (seed sequences, nucleotides 2–8) if their 5′ ends are changed or shifted. The interesting expression bias may implicate functional selection: identities of 3′ isomiRs were not changed and ensured the same “seed sequences” to bind target mRNAs and regulate biological processes. On the other hand, 3′ addition events further enriched dominant 3′ isomiRs (Figure 1, Figure 2 and Figure 5). Some enzymes have been associated with the 3′ addition events in animals [23], [31], [41], [42], and adenosine was the most dominant and abundant additional nucleotide (Figure 1, Figure 2 and Figure 5) [23], [24]. Multiple isomiRs with end heterogeneity were not caused by RNA degradation during sample preparation steps [26], and were the final result of strict regulation during pre-miRNA processing. Taken together, these findings revealed isomiR spectrum should not be a random event due to imprecise and alternative cleavages of Drosha and Dicer during pre-miRNA processing, which might provide potential implication for biological processes, especially for complexity of isomiRs with 3′ non-template additional nucleotides. Therefore, we further performed global analysis of isomiR spectrum and expression distribution across normal and diseased samples.

Different miRNAs showed different type of isomiRs with various expression levels (Figure 5 and Figure 6). Relative expression levels of isomiRs from a given locus showed consistent or inconsistent distribution patterns across samples (Figure 5). Inconsistent expression distribution patterns, especially between normal and diseased samples, may indicate potential function and contribute to complex regulatory network. For example, isomiRs of hsa-miR-143 in severe sample showed different expression pattern with normal and mild samples, while isomiRs of hsa-miR-103 and hsa-miR-424 in normal sample showed inconsistent expression patterns with diseased samples (Figure 5). These miRNAs played a role in regulating important biological processes, such as cell growth and apoptosis, transcription according to experimentally validated target genes. Differentially expression pattern of isomiRs from a given locus may implicate potential regulation contribution, although we still have not experimental evidence. On the other hand, the most abundant isomiR spectrum might be different across normal and diseased samples despite these isomiRs were 3′ isomiRs and had the same “seed sequences” (Figure 5 and Table S4). Indeed, the most abundant isomiR may be different among different species and even different type of samples from the same species [26], here we firstly found the dominant isomiR varies in placental samples with normal control and different degrees of pre-eclampsia (Table S4). For example, hsa-miR-519d showed two different dominant sequences in normal and diseased samples, and they were inconsistent with canonical hsa-miR-519d. Nonetheless, these dominant sequences had the same 5′ ends and “seed sequences” with the canonical sequence, and only lengths and 3′ ends were changed (Table S4). It is uncertain that difference of 3′ ends and lengths might play a role in development of pre-eclampsia or be associated with activity of miRNAs. Further studies, especially experimental studies, should reveal whether 3′ ends and lengths influence activity of miRNAs, as well as to elucidate potential mechanisms of miRNAs with 3′ additions during regulating biological processes.

Overall, multiple isomiRs should not be a random result due to imprecise and alternative cleavage of Drosha and Dicer during pre-miRNA processing. The variety of isomiRs may play a role in regulating biological processes and reveal potential biological implication. The major reasons are as follows. (1) Type of isomiRs may be different across different miRNAs, and no strict correlation was detected between type of isomiRs and expression level of miRNA; (2) Most isomiRs were 3′ isomiRs with 3′ variations due to bias of cleavage, and these 3′ isomiRs always ensured the same identities with their canonical miRNAs; (3) Generally, 1–3 abundant isomiRs were identified from a given locus, but number of abundant isomiRs and dominant cleavage sites showed diversity across different miRNAs. Abundant isomiRs always were 3′ isomiRs and had the same 5′ ends and “seed sequences”, but none of them was detected with 3′ non-template additional nucleotides; (4) 3′ addition event was a widespread phenomenon. Additional nucleotide showed a strong bias towards adenosine. Modified isomiR also could be abundantly expressed despite it was not characterized as abundant isomiR from a specific miRNA locus; (5) The most abundant spectrum may be different in the same tissues from normal and diseased samples, even from samples with different degrees of disease. Despite different sequences were detected, they were 3′ isomiRs and had the same identities; and (6) IsomiR spectrums and their expression distributions always were stability, and their alternation may influence specific biological processes, even may contribute partly to pathogenesis of disease.

### Consistent differential expression profiles based on different estimation methods

xmiRNAs always showed 1–3 abundant isomiRs and other rare isomiRs, and expression levels of miRNAs might show different distribution patterns based on estimation methods of the most abundant isomiR and sum of all isomiRs, respectively [40]. In the study, we collected the top 10 abundant miRNA species based on the most abundant isomiR, but they showed inconsistent distributions according to estimation method of sum of all isomiRs (Table 1). Due to varieties of type and expression levels of isomiRs, especially number of abundant isomiRs, expression distributions indicated different patterns according to different estimation methods. As expected, the top 10 abundant miRNAs showed different miRNA species and distributions (Figure 3A and Figure 3B).

Based on the inconsistent distributions, we then asked whether there were inconsistent differential expression profiles based on different estimation methods across samples. Here, despite detailed fold change values were different, consistent differentially expressed miRNAs were collected (default fold change values were more than 4 or lower than −4) across samples according to the most abundant isomiR and sum of all isomiRs, respectively (Figure 4A and Figure 4B). The consistent differential expression profiles were mainly resulted from conservation of isomiR spectrum across different samples, although different expression patterns were detected. In fact, isomiR spectrum was also conserved across different species [26]. Therefore, consistent differentially expressed miRNA profiles would be obtained based on either sequence count of the most abundant isomiR or sum of all isomiR sequence counts. Additionally, experimental miRNA research is prone to detect abundant isomiRs, particularly involved complexity of isomiRs with 3′ additions, perhaps the most abundant isomiR looks like a practical marker to profile miRNAs. Simultaneously, miRNA with 3′ addition, actual specific isomiR with 3′ non-template additional nucleotide, was not abundant isomiR from the specific miRNA locus. These modified miRNAs have potential function to influence miRNA stability and play a role in interactions of miRNA:target [23], [24]. Therefore, in the study, at isomiR level, we also assessed differentially expressed isomiRs with 3′ additions based on their sequence counts (discussed later).

### Potential critical roles of isomiRs with 3′ additions

Increasing evidence has demonstrated that miRNAs are subject to 3′ nucleotide additions, especially post-transcriptional non-template 3′ additions of adenosines or uridines [4], [14], [17], [22], [23], [24], [25], [26], [27], [28], [29], [30]. Here, we attempted to find potential relationship between modified isomiRs and human disease by performing a comprehensive analysis based on high-throughput sequencing datasets. Over 30% isomiRs were detected with 3′ additions, while they showed lower (<15%) expression percentage (Figure 1A and Figure 1B). Similar to recent studies [23], [24], adenosine was the most abundant and prevalent 3′ additional nucleotide (Figure 1C–F). Although many isomiRs were detected the phenomenon of 3′ addition events, they always showed lower expression levels (percentage was less than 17%) and were not abundant isomiRs (Figure 2 and Figure 5). According to the fact that many isomiRs were characterized as modified isomiRs but showed lower expression levels (<15%), negative or ambiguous 3′ additions from errors introduced during small RNA library preparation contribute partly to the widespread phenomenon. Indeed, we also assessed the 3′ addition events based on abundant isomiRs (sequence counts >99), and we found only about 20% of total isomiRs were detected the phenomenon (Figure S4). Generally, these modified isomiRs had lower percentage of total expression from a given miRNA locus, although other isomiRs from the same locus were highly expressed (for example hsa-miR-24, Figure 2 and Figure 5). However, some of these modified isomiRs also were unexpectedly quite abundant, which suggests a potential role in regulatory network. 3′ addition events play a role in modulating miRNA effectiveness and stability or strengthen miRNA:target interactions [23], [24]. We then asked whether it also contributes to pathogenesis of human disease. Systematic analysis showed that isomiRs with 3′ additions in normal sample was more prevalent than diseased samples (Figure 1 and Figure S4). Compared with miRNA level, modified isomiRs showed more private isomiRs based on the top 10 abundant species, and some of them were derived from the same miRNA locus (Figure 3). Many modified isomiRs were abundantly expressed and had higher expression levels, although they showed lower relative percentage of total expression and were not abundant isomiRs in the specific miRNA locus. These findings revealed potential role of 3′ addition events in pathogenesis of pre-eclampsia.

Generally, isomiRs with 3′ additions were not abundant isomiR species from a given locus, although in some cases they also showed higher sequence counts (Figure 2 and Figure 5). The non-template 3′ additional nucleotides are added after Dicer processing in animals [24], [31], and these additional nucleotides are added to 3′ ends of miRNAs that might be canonical miRNAs, shorter or longer miRNAs (Figure 2). The additional nucleotide selection is not random. For example, hsa-miR-24 had several modified isomiRs, but adenosines and uridines were dominant additional nucleotides (Figure 2A). These results revealed that non-random addition event might play critical role in miRNA regulatory network. In fact, we found a distinct subset of differentially expressed modified isomiRs, especially for isomiRs with 3′ adenosines (Figure 4C). Interestingly, these isomiRs might not be derived from differentially expressed miRNAs (Figure 4). Almost all of them had the same 5′ ends with their canonical miRNAs. Therefore, we subsequently collected experimentally validated miRNA-target interactions of these miRNAs from the miRTarBase database [43], and further Gene Ontology analysis revealed enrichment for specific biological process categories, including regulation of transcription, apoptosis, cell cycle, immune response, response to stimulus, etc. Furthermore, some double-nucleotide unambiguous 3′ non-template additions, including AA and UU additions, were observed in literature [23]. Here we also found the interesting double additional nucleotides, especially for dominant AA, AU and GA (Figure S5). Similarly, expression difference could be detected among samples, especially between normal and diseased samples (Figure S5). As an important post-transcriptional processing event, isomiRs with 3′ additions may play a role in attenuating effectiveness of specific miRNA by interfering with incorporation into RISC (the RNA-induced silencing complex), similar to “miRNA assassins” [23], [44].

Taken together, isomiRs with 3′ non-template additional nucleotides may be involved in pathogenesis of human disease. The phenomenon of 3′ addition events may influence miRNA stability and play a role in interactions of miRNA:target [23], [24], and we therefore believe further studies will provide more detailed potential relationship between these abundant modified isomiRs and human diseases. Specific isomiR with 3′ additional nucleotide will be a new marker to discover mechanism of human diseases.

## Materials and Methods

### Small RNA library preparation and sequencing

Placental samples of pregnant women were obtained from Zhongda Hospital, Nanjing, China. The institutional review board at Zhongda Hospital approved the tissue acquisition protocol to conduct the study. Written informed consent was obtained from each participant before tissue acquisition. Of these samples, one of them was from normal control pregnant woman, and others were from pregnant women diagnosed as mild and severe pre-eclampsia, respectively. Total RNAs of these samples were extracted with TRIzol (Invitrogen). Small RNAs were isolated from their total RNAs using mirVana™ miRNA Isolation Kit (Ambion). According to the protocol of SOLiD™ Small RNA Expression Kit (Life Technologies), purified small RNAs were subjected to miRNA library construction. Sequencing was carried out using SOLiD™ sequencing platform (ABI, Life Technologies) at the State Key Laboratory of Bioelectronics, School of Biological Science and Medical Engineering, Southeast University, China.

### Small RNA analysis

Sequencing files in colorspace were collected from SOLiD System based on a two-base encoding technology. According to SOLiD miRNA analysis pipeline (http://SOLiDsoftwaretools.com/gf/project/srna/), human other non-coding RNAs (ncRNAs, such as tRNAs, rRNAs, snoRNAs, snRNAs, etc.) were firstly filtered. The remaining sequencing reads were then analyzed by aligning to known human miRNA precursor sequences from the miRBase database (Release 16.0, http://www.mirbase.org/) [45] using Bowtie 0.12.7 [46]. Only one mismatch was allowed without considering adaptor sequences. Due to employed a two-base encoding technology, only one single mismatch always was a sequencing error and should be corrected according to reference nucleotide. If the position of the mismatch was located at 3′ end, and followed colorspace (in fact, it is the first colorspace of adaptor sequence) was also a mismatch, it should be a non-template additional nucleotide or 3′ modification event. Based on these, 3′ nucleotide additions of miRNAs were identified and collected.

### Assessment of differentially expressed miRNA profile

Due to multiple isomiRs were yielded from a single miRNA locus, miRNA profile was assessed by using sequence count of the most abundant isomiR and sum of all isomiRs sequence counts, respectively. Simultaneously, we also estimated differentially expressed miRNAs based on the different methods. Differentially expressed isomiRs with 3′ non-template additional nucleotides were also estimated and selected between pairwise samples. Furthermore, isomiR spectrum and expression distribution pattern from a given locus were analyzed across the three samples.

### Gene Ontology enrichment analysis

We selected those differentially expressed isomiRs with 3′ additions and collected their experimentally validated target sites from the miRTarBase database [43] if they had the same “seed sequences” with their canonical miRNA sequences in the miRBase database. These genes were then queried for Gene Ontology Enrichments using CapitalBio® Molecule Annotation System V4.0.

## Supporting Information

Figure S1 Length distribution of miRNAs through analyzing deep sequencing datasets.
(TIF)

Figure S2 **Frequencies of heterogeneity of 5′ and 3′ ends.** The frequency is estimated based on type of isomiRs without involved isomiRs with 3′ additions. 3′ isomiRs are quite prevalent than 5′ isomiRs across the three samples.
(TIF)

Figure S3 **Distribution patterns of miRNAs and type of isomiRs.** Expression distributions of miRNAs are assessed from lower to higher expression levels based on sum of all isomiR sequence counts, while their corresponding types of isomiRs show chaos distributions. No strict correlation is found between expression level of miRNA and its type of isomiRs. All of these miRNAs are abundantly expressed in corresponding sample (sequence count of the most abundant isomiR is over 999). Type of isomiRs is assessed based on their sequence counts (>99). To avoid great expression difference among miRNAs, some miRNAs with quite high expression levels are removed.
(TIF)

Figure S4 **Percentage distributions of 3′ additions across different samples.** Here we only consider those isomiRs that sequence counts are over 99. Percentage of 3′ additions based on (A) all type of isomiRs; (B) sequence counts of all isomiRs; (C) type of all isomiRs; (D) sequence counts of all isomiRs; (E) type of isomiRs with 3′ additions; (F) sequence counts of isomiRs with 3′ additions.
(TIF)

Figure S5 The 3′ non-template double additional nucleotides and their percentage across different samples.
(TIF)

Table S1 The number of total sequencing reads and reads that match to known miRNAs.
(DOC)

Table S2 Differentially expressed miRNAs with 3′ additions and their experimental validated gene targets from the miRTarBase database.
(DOC)

Table S3 Sequence count of miRNA (based on the most abundant isomiR) and its type of isomiRs across different samples.
(DOC)

Table S4 The most abundant isomiR sequence varies among different samples.
(DOC)
