# Supplementary figures and images for: A Comprehensive Survey of miRNA Repertoire and 3′ Addition Events in the Placentas of Patients with Pre-Eclampsia from High-Throughput Sequencing
Source: PLoS One. 2011 Jun 22;6(6):e21072. doi: 10.1371/journal.pone.0021072 (PMC3120834; doi:10.1371/journal.pone.0021072)

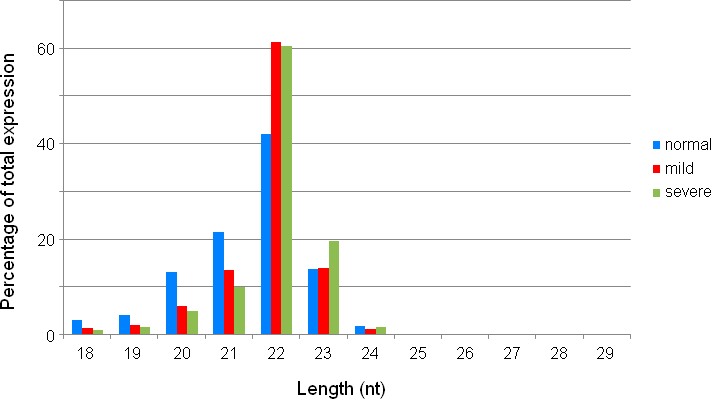

Supplement: Figure S1 — Length distribution of miRNAs through analyzing deep sequencing datasets. (TIF) [file pone.0021072.s001.tif]

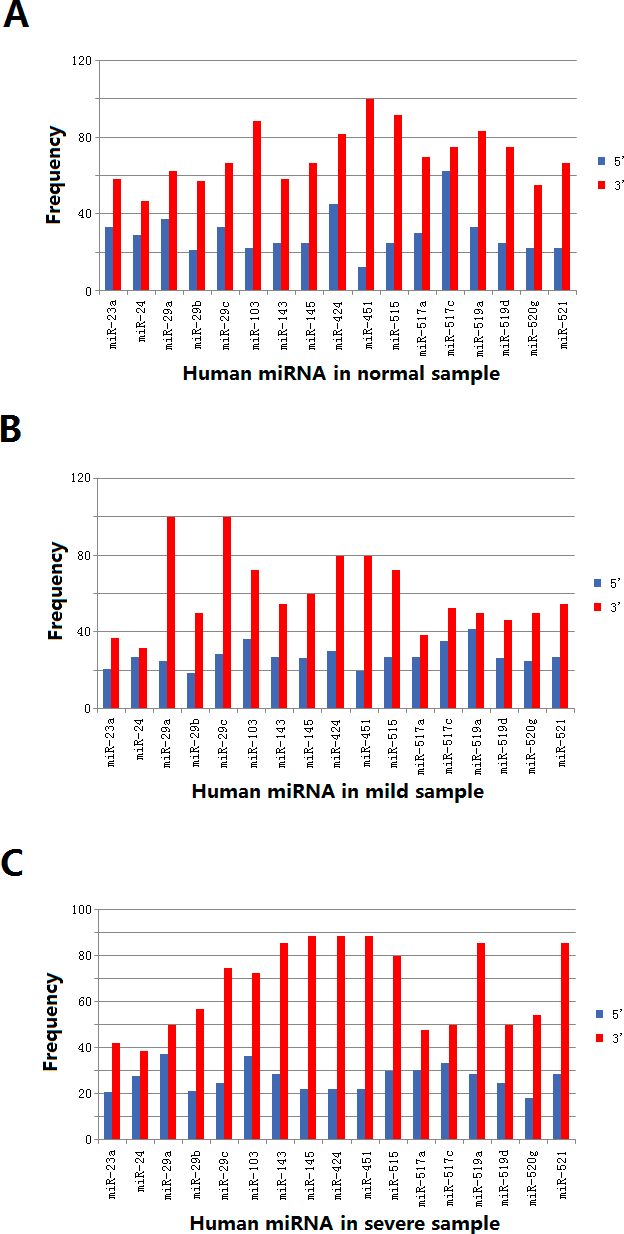

Supplement: Figure S2 — Frequencies of heterogeneity of 5′ and 3′ ends. The frequency is estimated based on type of isomiRs without involved isomiRs with 3′ additions. 3′ isomiRs are quite prevalent than 5′ isomiRs across the three samples. (TIF) [file pone.0021072.s002.tif]

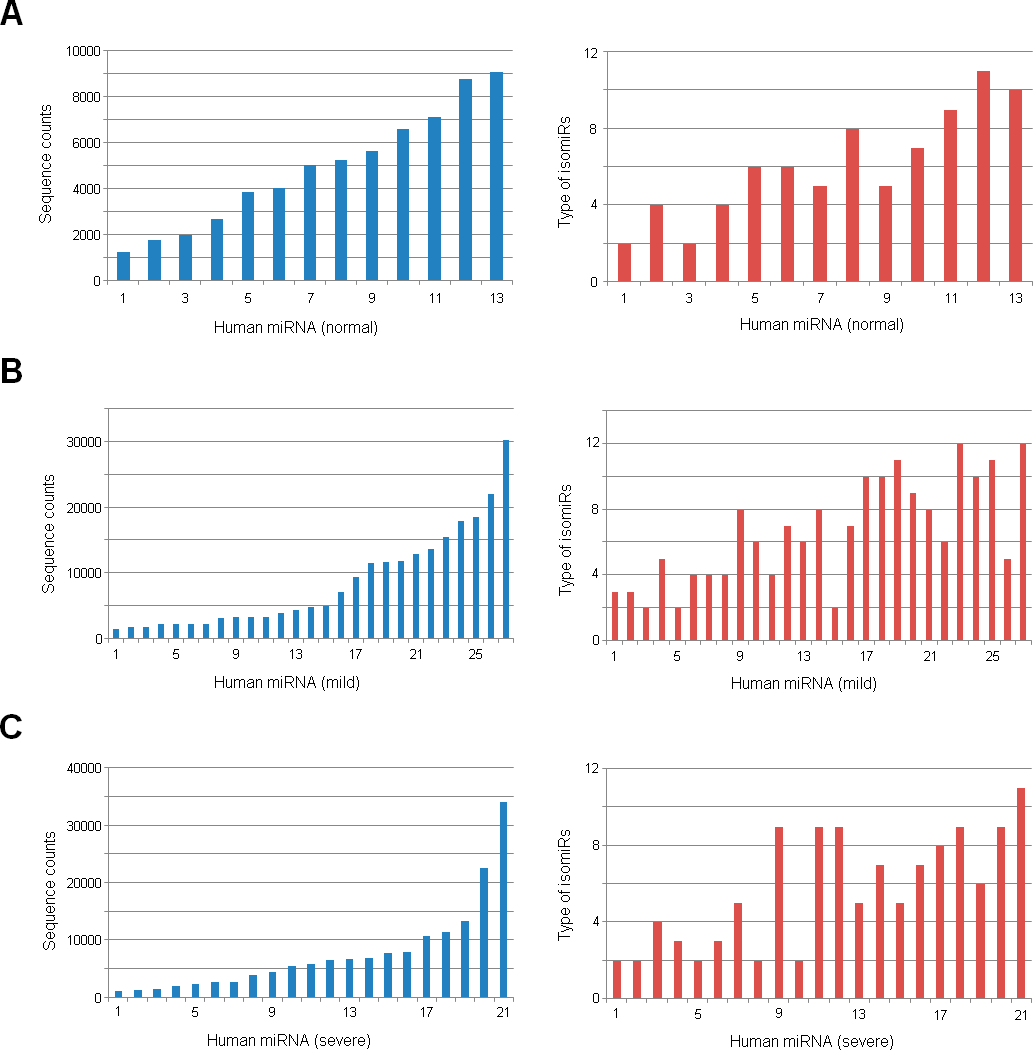

Supplement: Figure S3 — Distribution patterns of miRNAs and type of isomiRs. Expression distributions of miRNAs are assessed from lower to higher expression levels based on sum of all isomiR sequence counts, while their corresponding types of isomiRs show chaos distributions. No strict correlation is found between expression level of miRNA and its type of isomiRs. All of these miRNAs are abundantly expressed in corresponding sample (sequence count of the most abundant isomiR is over 999). Type of isomiRs is assessed based on their sequence counts (>99). To avoid great expression difference among miRNAs, some miRNAs with quite high expression levels are removed. (TIF) [file pone.0021072.s003.tif]

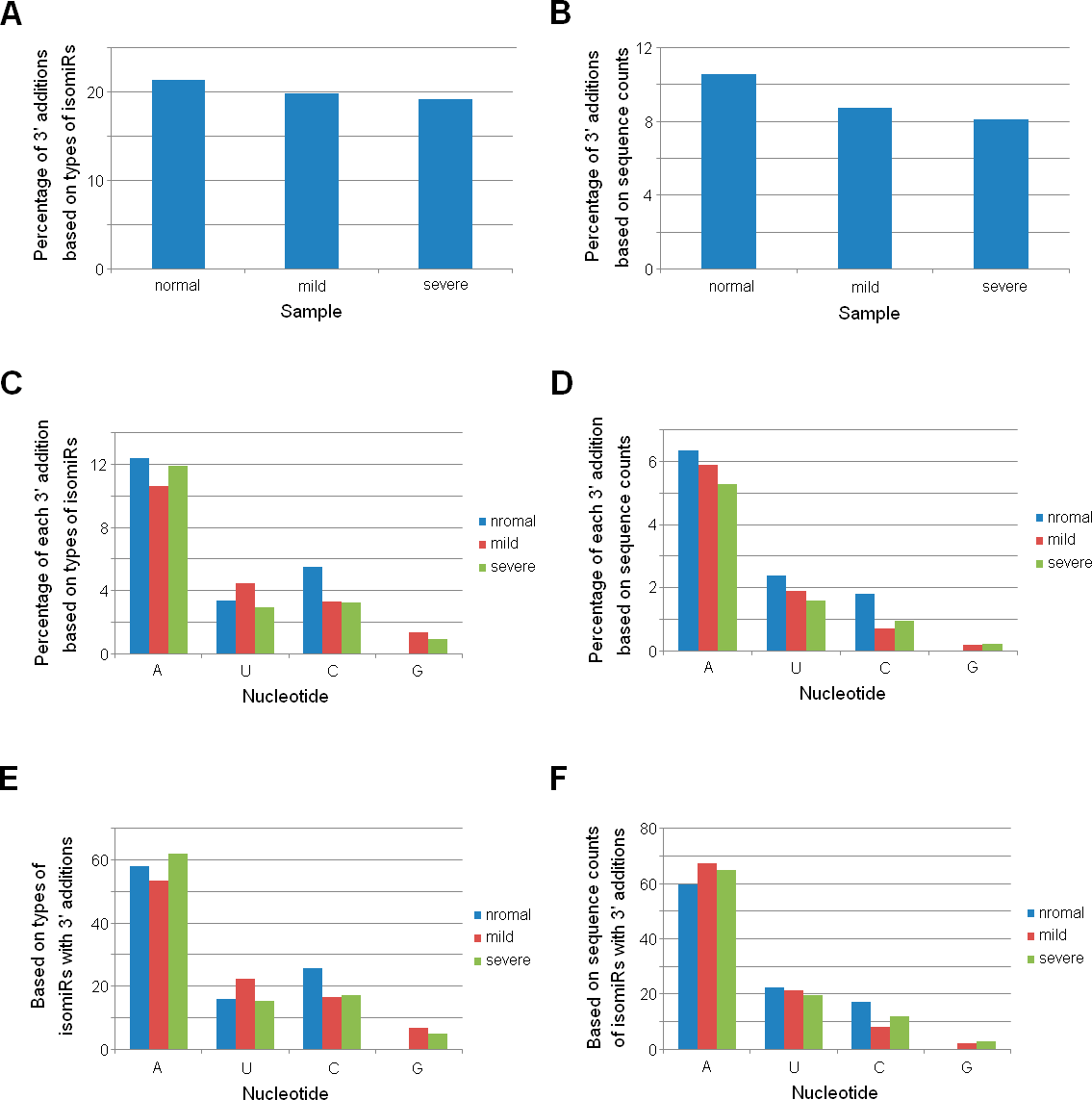

Supplement: Figure S4 — Percentage distributions of 3′ additions across different samples. Here we only consider those isomiRs that sequence counts are over 99. Percentage of 3′ additions based on (A) all type of isomiRs; (B) sequence counts of all isomiRs; (C) type of all isomiRs; (D) sequence counts of all isomiRs; (E) type of isomiRs with 3′ additions; (F) sequence counts of isomiRs with 3′ additions. (TIF) [file pone.0021072.s004.tif]

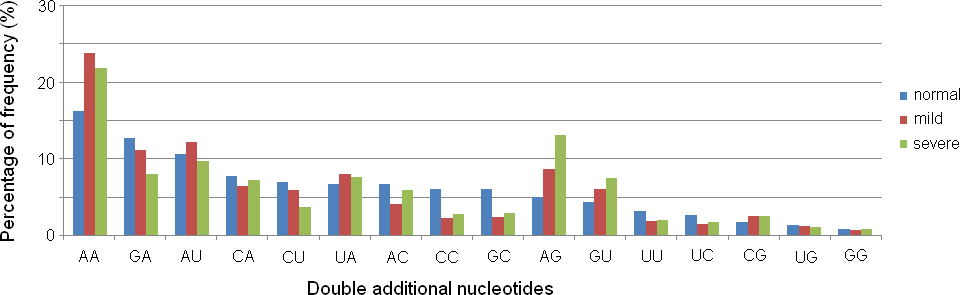

Supplement: Figure S5 — The 3′ non-template double additional nucleotides and their percentage across different samples. (TIF) [file pone.0021072.s005.tif]
