# Supplementary material for: A Comprehensive Survey of miRNA Repertoire and 3′ Addition Events in the Placentas of Patients with Pre-Eclampsia from High-Throughput Sequencing
Source: PLoS One. 2011 Jun 22;6(6):e21072. doi: 10.1371/journal.pone.0021072 (PMC3120834; doi:10.1371/journal.pone.0021072)
Supplement: Table S1 — The number of total sequencing reads and reads that match to known miRNAs. (DOC) [file pone.0021072.s006.doc]

**Table S1.** **The number of total sequencing reads and reads that match to known miRNAs.**

| **Sample** | **Total reads without adaptors** | **Number of reads matched to known miRNAs (0 mismatch)** | **Number of reads matched to known miRNAs (1 mismatch)** | **Number of reads matched to known miRNAs (2 mismatch)** |
| --- | --- | --- | --- | --- |
| normal | 543,884 | 75,301 | 139,034 | 239,668 |
| mild | 1,291,438 | 348,198 | 535,539 | 795,976 |
| severe | 857,440 | 194,305 | 318,249 | 483,544 |
