# Supplementary material for: A Comprehensive Survey of miRNA Repertoire and 3′ Addition Events in the Placentas of Patients with Pre-Eclampsia from High-Throughput Sequencing
Source: PLoS One. 2011 Jun 22;6(6):e21072. doi: 10.1371/journal.pone.0021072 (PMC3120834; doi:10.1371/journal.pone.0021072)
Supplement: Table S2 — Differentially expressed miRNAs with 3′ additions and their experimental validated gene targets from the miRTarBase database. (DOC) [file pone.0021072.s007.doc]

**Table S2.** **Differentially expressed miRNAs with 3’ additions and their experimental validated gene targets from the miRTarBase database.**

| **miRNA** | **Differentially expressed between:** | **Experimental validated target genes** |
| --- | --- | --- |
| hsa-miR-126* | mild & normal | SLC45A3 |
| hsa-miR-143 | severe & normal  severe & mild | DNMT3A, Elk-1, ERK5, FNDC3B, Klf4, KRAS, MYO6 |
| hsa-miR-24 | mild & normal  severe & normal | ALK4, AURKB, CCNA2, CDC2, CDK4, DHFR, E2F2, FEN1, HNF4A, KIAA0152, L protein, MAPK14, MYC, NOTCH1, P protein,  p16, TRIB3 |
| hsa-miR-29a | mild & normal  severe & mild | B7-H3, BACE1, BCL2, CDK6, col4a1, col4a2, CXXC6, Dkk1, DNMT3A, DNMT3B, Kremem2, MCL-1, MCL1, nef, RAN, sFRP2, TTP |
| hsa-miR-30d | severe & mild | GNAI2 |
| hsa-miR-424 | mild & normal  severe & normal | ANLN, ATF6, CCND1, CCND3, CCNE1, CCNF, CDC14A, CDC25A, CDK6, CHEK1, FGFR1, ITPR1, KIF23, MAP2K1, PIAS1, PLAG1, WEE1 |
| hsa-miR-519d | mild & normal  severe & normal | CDKN1A, PPARA |

All of these isomiRs have the same 5’ ends and “seed sequences” with their canonical miRNA sequences in the miRBase database.
