# Supplementary material for: A Comprehensive Survey of miRNA Repertoire and 3′ Addition Events in the Placentas of Patients with Pre-Eclampsia from High-Throughput Sequencing
Source: PLoS One. 2011 Jun 22;6(6):e21072. doi: 10.1371/journal.pone.0021072 (PMC3120834; doi:10.1371/journal.pone.0021072)
Supplement: Table S3 — Sequence count of miRNA (based on the most abundant isomiR) and its type of isomiRs across different samples. (DOC) [file pone.0021072.s008.doc]

**Table S3.** **Sequence count of miRNA (based on the most abundant isomiR) and its type of isomiRs across different samples.**

| **miRNA** | **normal (count-type)** | **mild (count-type)** | **severe (count-type)** |
| --- | --- | --- | --- |
| miR-100 | 831-4 | 2031-4 | 948-4 |
| miR-103 | 3797-5 | 8242-11 | 8772-8 |
| miR-107 | 570-2 | 1102-8 | 1005-4 |
| miR-125a | 404-1 | 1655-2 | 833-1 |
| miR-126* | — | 1614-6 | 153-1 |
| miR-130a | — | 4796-2 | 5386-2 |
| miR-141 | — | 463-2 | 1145-2 |
| miR-143 | 3440-9 | 1097-4 | 229-2 |
| miR-145 | 2779-11 | 3816-10 | 2400-9 |
| miR-17 | 266-1 | 1205-4 | 834-2 |
| miR-191 | 824-4 | 2828-6 | 1360-3 |
| miR-199a-5p | 777-3 | 1669-7 | 604-3 |
| miR-21 | 639-2 | 622-2 | 2143-2 |
| miR-22 | 1168-2 | 268-1 | 278-1 |
| miR-223 | — | 1104-5 | 982-5 |
| miR-23a | 2223-6 | 9136-11 | 4950-9 |
| miR-23b | 1892-4 | 4569-10 | 2378-9 |
| miR-24 | 14488-9 | 123823-18 | 80011-17 |
| miR-29a | 966-2 | 579-1 | 6660-5 |
| miR-29b | 3981-8 | 3472-8 | 2156-5 |
| miR-29c | 1912-2 | 324-1 | 3768-2 |
| miR-30b | 368-3 | 1281-3 | 758-1 |
| miR-30d | 490-3 | 1735-4 | — |
| miR-424 | 3492-7 | 1972-2 | 1235-2 |
| miR-451 | 1506-6 | 5892-10 | 2385-7 |
| miR-515 | 3688-10 | 4168-8 | 3211-7 |
| miR-517a | 173-1 | 70581-17 | 27371-11 |
| miR-517c | 121-1 | 10273-6 | 5142-5 |
| miR-518b | 1395-4 | 1390-3 | — |
| miR-518e | — | 3105-7 | — |
| miR-519a | 744-1 | 20513-5 | 12136-6 |
| miR-519d | 748-6 | 16231-12 | 15031-9 |
| miR-520g | 644-9 | 6374-12 | 3306-9 |
| miR-521 | 4551-5 | 9355-9 | 2498-3 |

miRNAs are presented here if their sequence counts (the most abundant isomiR) are over 999 at least in a sample. Type of isomiRs only contains those isomiRs with higher counts (> 99). “—” shows the miRNA is not detected or has lower expression levels (less than 100) in the sample.
