# Supplementary material for: A Comprehensive Survey of miRNA Repertoire and 3′ Addition Events in the Placentas of Patients with Pre-Eclampsia from High-Throughput Sequencing
Source: PLoS One. 2011 Jun 22;6(6):e21072. doi: 10.1371/journal.pone.0021072 (PMC3120834; doi:10.1371/journal.pone.0021072)
Supplement: Table S4 — The most abundant isomiR sequence varies among different samples. (DOC) [file pone.0021072.s009.doc]

**Table S4. The most abundant isomiR sequence varies among different samples.**

| **miRNA** | **Samples** | **Sequence of the most abundant isomiR** | **Fold** | **miRBase sequence** |
| --- | --- | --- | --- | --- |
| miR-145 | normal | GUCCAGUUUUCCCAGGAAUCC | 1.62 | GUCCAGUUUUCCCAGGAAUCCCU |
|  | mild | GUCCAGUUUUCCCAGGAAUCCCU | 1.52 |  |
|  | severe | GUCCAGUUUUCCCAGGAAUCCCU | 2.15 |  |
| miR-451 | normal | AAACCGUUACCAUUACUGAGUU | 1.15 | AAACCGUUACCAUUACUGAGUU |
|  | mild | AAACCGUUACCAUUACUGAGU | 1.18 |  |
|  | severe | AAACCGUUACCAUUACUGAGUUU | 1.06 |  |
| miR-515 | normal | GAGUGCCUUCUUUUGGAGCGUU | 1.61 | GAGUGCCUUCUUUUGGAGCGUU |
|  | mild | GAGUGCCUUCUUUUGGAGCGUUA | 1.22 |  |
|  | severe | GAGUGCCUUCUUUUGGAGCGUUA | 1.59 |  |
| miR-519d | normal | CAAAGUGCCUCCCUUUAGAGU | 2.78 | CAAAGUGCCUCCCUUUAGAGUG |
|  | mild | CAAAGUGCCUCCCUUUAGAGUGU | 2.53 |  |
|  | severe | CAAAGUGCCUCCCUUUAGAGUGU | 5.19 |  |

Although the most abundant isomiR varies among different placental samples, these isomiRs were 3’ isomiRs with various 3’ ends but with the same 5’ ends and “seed sequences”. The most abundant isomiR may be consistent or inconsistent with canonical miRNA sequence. To understand expression difference between various isomiRs, we also estimated fold change of the most and secondary abundant isomiRs (Fold). Hsa-miR-519d is an evident miRNA that shows different dominant sequence across normal and diseased samples.
